# Orphan Drug Pricing: An Original Exponential Model Relating Price to the Number of Patients

**DOI:** 10.3390/scipharm84040618

**Published:** 2016-01-24

**Authors:** Andrea Messori

**Affiliations:** HTA Unit, Regional Health Service, ESTAR, via San Salvi 12, 50135 Firenze, Italy; andrea.messori@estar.toscana.it; Tel.: +39-338-9513583

**Keywords:** price determination, orphan drugs, eculizumab, bosentan

## Abstract

In managing drug prices at the national level, orphan drugs represent a special case because the price of these agents is higher than that determined according to value-based principles. A common practice is to set the orphan drug price in an inverse relationship with the number of patients, so that the price increases as the number of patients decreases. Determination of prices in this context generally has a purely empirical nature, but a theoretical basis would be needed. The present paper describes an original exponential model that manages the relationship between price and number of patients for orphan drugs. Three real examples are analysed in detail (eculizumab, bosentan, and a data set of 17 orphan drugs published in 2010). These analyses have been aimed at identifying some objective criteria to rationally inform this relationship between prices and patients and at converting these criteria into explicit quantitative rules.

## 1. Introduction

The price of orphan drugs is generally higher than that determined according to value-based principles [1]. However, the increase in price for these agents is not presently governed through any specific methodology. Although a number of factors have been recognized to influence this price increase, the decisions in this area remain largely subjective. Briefly, disease prevalence is the most well-known factor implicated in the pricing process of orphan drugs [1,2,3], while the role of other factors is still a matter of controversy.

Previous reports [2,3] focused on orphan drugs have explored the mathematical relationship to link disease prevalence with prices. In particular, a preliminary equation based on an exponential decay in which prices are reduced as disease prevalence increases, has been described.

In this report, an improved mathematical model that employs an innovative conceptual framework for drug pricing has been developed and in the case of orphan drugs, it suggests a price value as a function of disease prevalence.

This conceptual framework for orphan drugs has the same exponential design as that previously proposed for high budget-impact drugs (e.g., sofosbuvir [4], evolocumab [5], alirocumab [5], ranibizumab [6]), in which these expensive drugs typically employed in large patient populations are priced through price-volume agreements that reduce the price as more patients are being treated.

## 2. Description of the Exponential Model

PRICE = f(Npt) = fPRICE × e^(0.693/PDP) x (1000 − Npt)^ (1)

where:

- Npt is the expected number of treated patients;
- PRICE (in euro/patient) is the cost of the orphan drug treatment (expressed as a function of Npt) which is assumed to undergo an exponential increase as Npt decreases;
- fPRICE (in euro) is the “baseline” price on the y axis attributed to the treatment (i.e., the full price with no discount) under the assumption that 1000 patients are treated; for the sake of simplicity, fPRICE has been set to 10,000 EUR in all examples described in this paper;
- PDP (expressed as the number of patients) is defined as the “price-doubling population” and in the framework of this exponential model, represents the decrease in the number of patients that iteratively determines a doubling of drug price.

This mathematical approach has been directly derived from standard pharmacokinetic modeling [7]. In pharmacokinetic modeling, the y-axis contains the values of drug concentration (that are handled as a function of time) and the x-axis is time. The present model instead has price on the y-axis (or better, the cost of treatment per patient) and the number of treated patients on the x-axis. Of course, there is no scientific interconnection between pharmacokinetic modelling and pharmacoeconomic modelling; however, mentioning the identity of these equations between pharmacoeconomics and pharmacokinetics is worthwhile because most pharmacologists are very familiar with these pharmacokinetic equations and pharmacoeconomics is increasingly being practiced by pharmacologists.

In the framework of this pricing model, drugs for which the number of treated patients exceeds 1000, though formally classified as orphan drugs, are handled as “normal” drugs that therefore are not associated with any increase in the price. Hence, the model described in this study actually refers to ultra-orphan drugs, a term that—in the context of our model—identifies agents indicated for less than 1000 patients on a nationwide basis.

This value of 1000 patients appears in the equation when the difference is calculated between 1000 and Npt. For simplicity reasons, this parameter of 1000 patients has been included as a constant in Equation (1); however, if necessary, values other than 1000 patients can be introduced in Equation (1) in replacement of 1000.

Our model is essentially a linear one. In fact, although the increase in price is exponential as the number of patients is reduced, applying a logarithmic transformation on the y-axis converts the curve of this relationship into a straight line.

**Figure 1:**
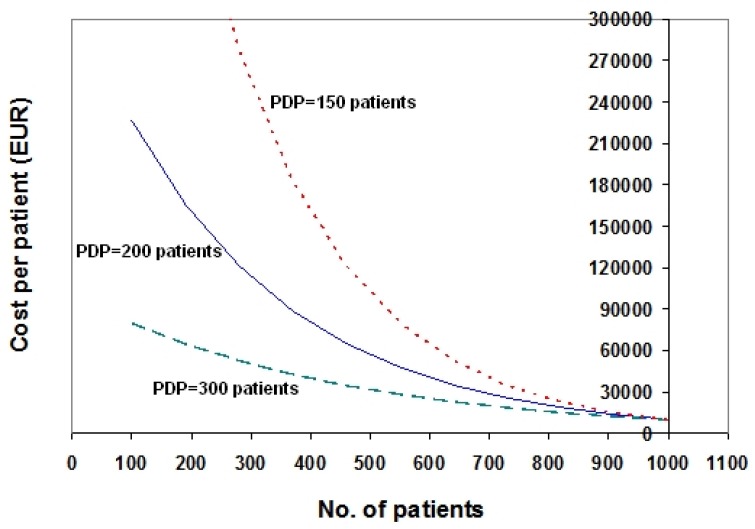
Price-vs.-patients relationship for orphan drugs. The graph shows three different exponential models that assume price-doubling population (PDP) = 150 patients (dotted line in red), 200 patients (solid line in blue), and 300 patients (dashed line in green), respectively. Other assumptions: full price (fPRICE) = 10,000 EUR.

The crucial point in the application of our model is the choice of a specific value of PDP (which is assumed to be applicable to the whole series of orphan drugs under examination). The graph shown in Figure 1 describes three different exponential models that assume PDP = 150 patients (dotted line in red), PDP = 200 patients (solid line in blue), and PDP = 300 patients (dashed line in green), respectively.

It is too early to say which of the values of PDP shown in Figure 1 can be considered the “best”. Other values not reported in Figure 1 could be appropriate as well. Obviously, this choice of a specific PDP value has important drug policy implications because the higher the value of PDP, the lower the economic incentive recognized to orphan drugs; and vice versa. Irrespective of the “extent” of this economic incentive (which is a matter of much debate [8,9,10,11,12,13]), one advantage of the present approach is that this incentive turns out to be the same for all orphan drugs, and this ensures more equity in handling different orphan agents within the same nationwide context.

## 3. Examples Based on Real Data

**Figure 2:**
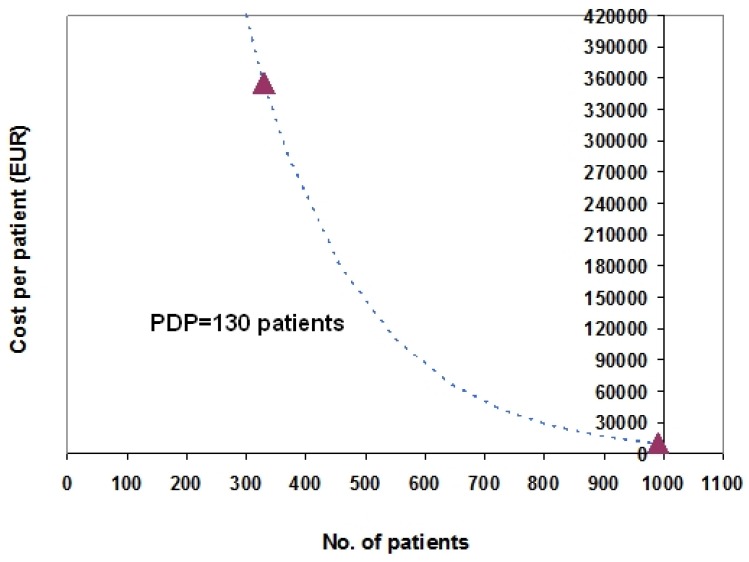
Price-vs.-patients relationship for orphan drugs. On the basis of a model assuming PDP = 130 patients (dotted line in blue), the graph shows the fitting procedure (two-point estimation) that allowed us to estimate PDP from the y-vs.-x of data pairs for eculizumab and bosentan (triangles). Eculizumab: patients = 330; cost per patient = 355,740 EUR; bosentan: patients = 990; cost per patient = 10,491 EUR. The mathematical calculations for estimating PDP are described in Appendix 1. Other assumptions: fPRICE = 10,000 EUR.

**Figure 3:**
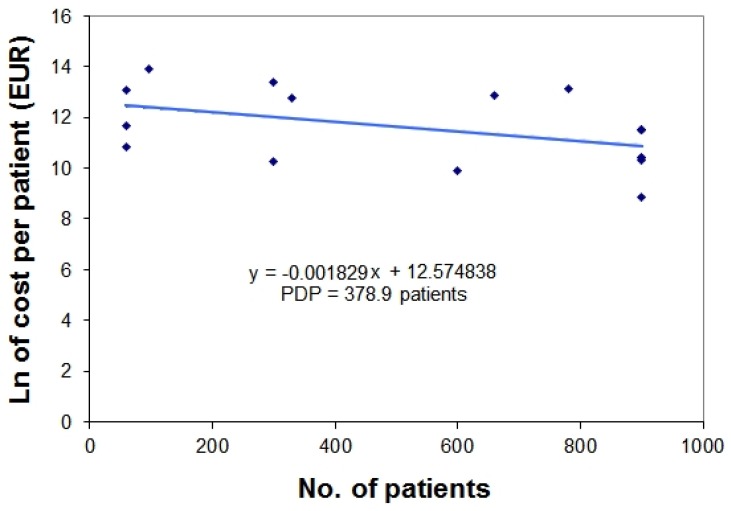
Price-vs.-patients relationship for a published data-set of 17 orphan drugs [2]. On the basis of 17 data pairs of price-vs.-patients (with log-transformed y-values), a linear regression is applied that identifies the linear regression equation shown in the graph. The slope of this equation is −0.001829 patients^-1^; hence, PDP is 378.9 patients. The numerical values of these 17 data pairs are reported in Appendix 2 along with information on the respective 17 orphan drugs.

This section presents an example of two-point fitting (Figure 2) in which PDP is estimated through a simple equation from the data of two orphan drugs (eculizumab and bosentan) and a more complex example (Figure 3) in which the same estimation is performed through a linear regression that analyses the data of 17 orphan drugs.

Data of Eculizumab and Bosentan: Two-Point Estimation of PDP (Figure 2). On the basis of a pre-specified model assuming PDP = 130 patients (dotted line in blue), the graph shown in Figure 2 shows the fitting procedure (two-point estimation) that allowed us to estimate PDP from the two y-vs.-x data pairs for eculizumab and bosentan (triangles). The mathematical calculations for estimating PDP are described in detail in Appendix 1. This example confirms that, when a pre-specified value of PDP is already known, the two-point fitting procedure can successfully estimate the correct PDP value (with a minimal estimation error).

Analysis of a Published Data Set of 17 Orphan Drugs (Figure 3). On the basis of 17 data pairs of price-vs.-patients (with log-transformed y-values) published by Fadda and Messori [3], a linear regression is calculated that identifies the equation of a line (Figure 3). The numerical values of these 17 data pairs are reported in Appendix 2 along with information on the respective 17 orphan drugs. The estimated slope of the linear equation is −0.001829 patients^−1^; hence, PDP is 378.9 patients.

## 4. Discussion

In using our exponential model, the most critical point is the choice of the value of PDP. It should be noted that, according to our model, all orphan drugs are parameterized on the basis of a common value of PDP; in other words, the values of PDP cannot be different for different orphan drugs, and so all agents included in an analysis are forced to be fitted to the same PDP value. This approach therefore determines a hoped-for homogeneity/standardization in the modeling.

On the other hand, one drawback of this standardization is that, at present, there are still no experiences in the determination of PDP. To our knowledge, the present study is the first worldwide experience that applies this exponential modeling approach. Much work in this area is therefore still needed.

According to the preliminary analyses described in this paper, the data shown in Figure 1 suggest a range of PDP values spanning from 150 to 300 patients. The two-point example shown in Figure 2 suggests a PDP of 130 patients. More importantly, the 17 data pairs shown in Figure 3 (representing a “true” data set that unfortunately dates back to year 2010) suggest a PDP of approximately 380 patients.

In general, the relationship between price and number of patients has a two-fold application. When prices are planned to increase as the number of patients decreases, the model acts in the framework of orphan drugs. So, the appropriate equation (i.e., Equation (1) described above) manages the orphanicity situations (with fewer than 1000 patients) and estimates the increase in price over the baseline value (i.e., fPRICE) when the number of patients (i.e., Npt) becomes less than 1000 patients. By contrast, when prices are planned to decrease as the number of patients increases, the model (with minimal differences; see References 4, 5, 6) acts in the framework of price-volume agreements. The appropriate equation (see Reference 4) manages these high budget-impact situations (generally with much more than 1000 patients; e.g., ranibizumab, sofosbuvir, etc.). In these situations, price is decreased as the number of patients (i.e., Npt) increases (and becomes greater -or much greater- than the baseline value set at about 1000 patients). In summary, orphan drugs are modeled on the basis of PDP while price-volume agreements are modeled on the basis of PHP (i.e., price halving population) [4,5,6].

The comparison between these two models underscores an advantage of this “unified” approach because the conceptual framework is essentially the same between PDP and PHP.

On the other hand, our study has several limitations. Firstly, our analyses referred to the pharmaceutical market of a country of 60 million people (like Italy). So, application of the same approach to other countries will obviously require some adaptations. How the model can be adapted to local situations remains a point open to future model improvements and to further original analyses.

In the past, the price-vs.-patients relationship for orphan drugs (despite the quantitative nature of the equations) has generally been applied on a purely empirical basis, i.e., in the absence of quantitatively defined, theoretical rules [1,8,9,10,11,12,13]. The experience described in this article represents the first attempt to start the construction of a sound theoretical framework in this field.

## Appendix 1

If the y-values of the price-vs.-patients relationship are subjected to a logarithmic transformation, the exponential model is converted into a linear model. This latter model is advantageous because PDP can easily be estimated from the “experimental data”, i.e., from the y-vs.-x data pairs. This estimation of PDP can either be based on the whole set of available y-vs.-x data pairs (in such a case the linear regression is calculated) or on a simpler two-point estimation. An example of this two-point estimation is described in this Appendix.

Let us consider the following two data-pairs (Figure 2):

-Eculizumab: patients = 330; cost per patient = 355,740 EUR -Bosentan: patients = 990; cost per patient = 10,491 EUR (2)

If the two values of cost are subjected to a logarithmic transformation, the following transformed y-values are obtained: eculizumab: y = 9.258273 (for 330 patients); bosentan: y = 12.78196 (for 990 patients).

The first-order rate constant of the model (which can be regarded as a slope) can be estimated from the difference in y-values divided by the difference in x-values. Hence:

first-order rate constant = (12.78196 − 9.258273)/(990-330) = 3.523682/660 = 0.00533891 patients^−1^

Finally,

PDP = 0.693/first-order rate constant = 0.693/0.00533891 patients^−1^ = 129.8 patients

In the model shown in Figure 2 (in which we have assumed that PDP is 130 patients), this example of a calculation shows that two data pairs can be adequate to reliably estimate PDP; in fact, on the basis of the two data pairs, PDP is estimated to be 129.8 patients.

## Appendix 2

This appendix reports, in Table A1, the numerical values of the 17 price-vs.-patients data pairs shown in Figure 3. These data pairs that were restricted to cases with fewer than 1000 patients were drawn from the paper by Fadda and Messori [2].

**Table A1:** Detailed information on the 17 data pairs of treatment cost-vs.-patients published by Fadda and Messori [2] for a series of orphan drugs §.

Orphan Drug	Nationwide Number of Treated Patients (per year) *	Yearly Treatment Cost per Patient (EUR)	Natural Logarithm of Treatment Cost **
mecasermin	60	50,000	10.81978
saproterin	60	116,637	11.66682
carglumic acid	60	489,206	13.10054
galsulfase	96	1,072,800	13.88578
deferasirox	300	28,470	10.25661
betaine anhydrous	300	649,335	13.3837
eculizumab	330	340,400	12.73788
icatibant	600	20,192	9.913042
alglucosidase alfa	660	396,000	12.88917
laronidase	780	504,000	13.13033
epoprostenol	900	100,000	11.51293
treprostinil	900	100,000	11.51293
sildenafil	900	6,928	8.843326
sitaxentan sodium	900	32,879	10.40059
bosentan	900	33,084	10.40681
iloprost	900	100,000	11.51293
ambrisentan	900	30,879	10.33783

§ Fadda and Messori [2] reported the data for other orphan drugs indicated for populations greater than 1000 patients; the data of these drugs were excluded from this analysis because our model does not recognize any increase in treatment cost when the number of patients exceeds 1000. * On the x-axis. ** On the y-axis.
